# Development of Merkel Cell Carcinoma in a Patient Receiving Rituximab

**DOI:** 10.1155/2022/1814338

**Published:** 2022-11-03

**Authors:** Bana Antonios, Ujjwal Karki, Kais Antonios, Bipin Ghimire, Mohammad Muhsin Chisti

**Affiliations:** ^1^Department of Internal Medicine, William Beaumont Hospital, Royal Oak, Michigan, USA; ^2^Department of Internal Medicine, St. Joseph Mercy Hospital, Ann Arbor, Michigan, USA; ^3^Department of Hematology & Oncology, William Beaumont Hospital, Royal Oak, Michigan, USA

## Abstract

Merkel cell carcinoma (MCC) is a rare, rapidly growing, and aggressive dermatological neoplasm. It is commonly reported in Caucasian ethnicities, and almost 50% of the patients have a concomitant malignancy and are on immunosuppressive chemotherapy. Here, we present a 79-year-old woman with a history of relapsed Stage II, grade III follicular lymphoma, receiving maintenance rituximab infusions. She presented with a raised erythematous papule on her left cheek. An excisional biopsy of the lesion confirmed a diagnosis of Merkel cell carcinoma. After which, she underwent a wider excision with 1-2 cm margins. PET scan did not reveal any FDG-avid uptake lesions that would be concerning for metastatic disease. However, she underwent a sentinel lymph node biopsy which was also negative. Thus, the diagnosis was finalized as Stage I (T1 N0 M0) MCC. There are only two reported cases in literature about the significant progression of Merkel cell carcinoma in patients who coincidentally were receiving rituximab as a part of treatment for another disease. This raises questions for future investigation and research on whether there is a direct association between rituximab use specifically and the rapid growth of MCC.

## 1. Introduction

Merkel cell carcinoma (MCC) is a rare and very aggressive skin malignancy, with a dramatic increase in incidence in the past few decades. It grows and metastasizes rapidly, and diagnosis in its early stages is often missed. Almost half of the patients with MCC have another active malignancy. Mostly reported are other cutaneous neoplasms, followed by leukemia and lymphomas [1–3].

MCC is mainly reported in the Caucasian population. Most affected patients are above the age of 50. Sun exposure is a major risk factor responsible for mutations created by ultraviolet-A radiation. In 2008, a novel discovery of Merkel cell polyomavirus (MCPyV) was detected in most patients with MCC that plays a role in oncogenicity [4, 5].

Most primary lesions are asymptomatic. Tumor size, depth, and lymphovascular invasion are important prognostic factors. Further imaging modalities (CT, MRI, and FDG-PET scans) are essential for staging. Treatment depends on the diagnosed stage. It varies from wide excision of the primary lesion followed by adjuvant radiation in early stages to systemic chemo and immunotherapy, [6–8].

## 2. Case Presentation

A 79-year-old woman with a history of relapsed Stage II, grade III follicular lymphoma was treated with four cycles of rituximab and bendamustine with an excellent response. She was subsequently placed on maintenance rituximab infusions every eight weeks. Six months later, she presented to the dermatology clinic with a newly raised erythematous papule on her left cheek. Excisional biopsy was consistent with a diagnosis of Merkel cell carcinoma (MCC). Pathology was positive for anti-cytokeratin 20 (CK20), AE1/AE3, CAM5.2, synaptophysin, and BerEp4 without significant expression of MART1. MCPyV was negative by immunohistochemistry and RNA in situ hybridization. She then underwent a wider resection around the primary lesion with 1-2 cm margins. PET scan did not show any FDG-avid uptake lymphadenopathy. However, due to the high complexity and aggressiveness of this malignancy, a decision was made to proceed with a sentinel lymph node biopsy which was also negative for regional spread. The final diagnosis was established as Stage I (T1, N0, and M0) MCC. Hence, neither adjuvant radiation therapy to the primary site, nor systemic treatment was indicated.

As her follicular lymphoma had been in remission for the past year, a shared medical decision was made to hold the rituximab infusions, due to the concern of very rare cases reporting an association between Merkel cell carcinoma and rituximab administration.

## 3. Discussion

Merkel cell carcinoma (MCC) is a rare cutaneous neuroendocrine cancer, first described by Cyril Toker in 1972. This aggressive malignancy has a high propensity for recurrence and metastasizes early to other organs. Its incidence has increased over the past two decades, notably at a significantly higher rate than other skin tumors like melanoma and most other solid tumors. Interestingly, there is an anticipated dramatic rise in MCC cases in the next few years, especially in patients over 65 years of age.

MCC is twice as frequent in males as in females, and most of the patients are of Caucasian ethnicity. More than half of the cases present with localized disease, whereas about less than 10% of patients are initially found to have distant metastasis. Unfortunately, advanced stages carry a high mortality rate, close to 33% within two years of diagnosis [1, 2].

It typically presents as a firm, nontender, flesh-colored, asymptomatic, and cutaneous nodule; ulceration and crusting are infrequent. However, these features are nonspecific, so most MCC cases are often clinically misdiagnosed as benign lesions.

It commonly affects sun-exposed areas, as ultraviolet (UV) radiation is a major risk factor. It is most commonly reported on the head and neck. Pathologically, Merkel cells express epithelial markers, such as cytokeratin CK20, which is usually stained in a paranuclear, dot-like pattern in almost all cases of MCC. Cytokeratin CK20 is highly sensitive and specific for Merkel cell carcinoma. It also helps distinguish MCC from other malignant cutaneous cancers such as melanoma, in which CK20 is negative. Expressions of stains such as inhibitor of apoptosis (IAP) have been reported to be associated with a worse outcome [3, 4].

The two major causative factors for MCC are Merkel cell polyomavirus (MCPyV-associated) and extensive UV exposure [5]. In 2008, a novel discovery of MCPyV was made; a nonenveloped, double-stranded DNA virus, detected in most MCC cases. MCPyV is a ubiquitous virus thought to be part of the human skin microbiome. Two major rare events need to occur within the same cell to induce oncogenicity. The viral genome must integrate into a host chromosome, and the large tumor (LT) antigen must be mutated and truncated, forming a functional oncoprotein, markedly smaller than the regular viral version of the protein [6]. Baseline assessment of the presence or absence of circulating antibodies to MCPyV oncoproteins at diagnosis is a useful prognostic indicator. Seronegative patients, such in our case, are almost 50% more likely to have a recurrence than seropositive patients. Thus, they may benefit from more frequent imaging surveillance. Monitoring seropositive patients with MCPyV antibody levels is an established surveillance method per the National Comprehensive Cancer Network (NCCN) guidelines; a rising antibody titer could indicate MCC recurrence, whereas a falling titer is highly reassuring [5].

Immunogenicity plays a key role in both virus-positive and virus-negative MCCs. Although the exact mechanisms by which immunosuppression affects the pathogenesis of MCPyV and UV radiation exposure remain unknown. It has been established that MCC occurs at a higher incidence and a younger median age in chronically immunosuppressed individuals, including organ transplant recipients, HIV-infected individuals, and those with hematological malignancies, such as our patient [3, 4]. The relationship between MCC and hematologic malignancies, especially B lymphoproliferative disorders, is well-recognized and was reported in a SEER-based study of over two million cancer patients by Howard et al. The study showed a significantly increased risk of developing MCC after any primary tumor and specifically after multiple myeloma, chronic lymphocytic leukemia, and non-Hodgkin lymphoma [9].

Our patient had relapsed Stage II, grade III follicular lymphoma that was successfully treated with four cycles of rituximab and bendamustine with an excellent response. Subsequently, she started maintenance rituximab infusions every eight weeks, and six months later, she was diagnosed with MCC, which fortunately, was caught at an early stage.

There have only been two reported cases of MCC following rituximab infusion. The first one was reported in 2002 in a 54-year-old male with follicular small cleaved lymphocytic lymphoma, who was placed on rituximab infusion after multiple lines of treatments. Ten months later, he presented with cervical lymphadenopathy and hepatomegaly, and a biopsy confirmed MCC, whereas his lymphoma was in remission. The second one was reported in 2006 in a 73-year-old female receiving rituximab infusions weekly for an acquired factor VIII inhibitor bleeding disorder. She then noted a cutaneous lesion on her cheek after the fourth infusion, which was biopsy-proven MCC [7, 8].

Treatment of MCC depends on the clinical stage and tumor features. In primary cutaneous MCC without evidence of locoregional spreading, wide surgical excision with 1-2 cm negative surgical margins can be sufficient. In patients with risk factors, such as chronic immunosuppressive state or lesion size >1 cm, such as in our patient, a sentinel lymph node biopsy (SLNB) is recommended to determine the need for adjuvant radiation therapy.

Our patient's SNLB was negative. Therefore, the decision was made to continue clinical observation and follow up every 3-6 months, for the first three years after diagnosis, based on the NCCN guidelines.

In the era of immunotherapy, immune checkpoint inhibitors (ICIs) are the cornerstone of therapy. There have been clinical trials that support the use of avelumab, pembrolizumab, nivolumab, and ipilimumab. However, there has not been a study comparing the efficacy of one drug to another in the treatment of disseminated MCC. Chemotherapeutic agents can also be used in advanced stages, mainly platinum-based agents. Unfortunately, the response to it is usually brief [10, 11].

In conclusion, rituximab has become a widely used medication over the past two decades. Interestingly, during our literature review, we noted that the significant increase in MCC incidence occurred concurrently with the spreading use of rituximab for various oncological, rheumatological, and autoimmune disorders.

Whether or not to continue rituximab in such cases remains a big dilemma. Shared decision-making with our patient led to discontinuation of rituximab. Therefore, it is important to raise awareness of this clinical observation, which may prompt further investigations in the future to better assess the posssible association between rituximab and MCC. It would also trigger physicians and patients' attention to cautiously investigate any new skin lesion while they are on rituximab.
